# Current advances in temozolomide encapsulation for the enhancement of glioblastoma treatment

**DOI:** 10.7150/thno.82005

**Published:** 2023-05-08

**Authors:** Nerea Iturrioz-Rodríguez, Nicolas Sampron, Ander Matheu

**Affiliations:** 1 Cellular Oncology group, Biodonostia Health Research Institute, San Sebastian, Spain.; 2 IKERBASQUE, Basque Foundation for Science, Bilbao, Spain.; 3 Centro de Investigación Biomédica en Red de Fragilidad y Envejecimiento (CIBERfes), Carlos III Institute, Madrid, Spain.

**Keywords:** glioblastoma, temozolomide, encapsulation, nanosystems, targeted delivery

## Abstract

Glioblastoma is the most common and lethal brain tumor in adults. The incorporation of temozolomide (TMZ) into the standard treatment has increased the overall survival rate of glioblastoma patients. Since then, significant advances have been made in understanding the benefits and limitations of TMZ. Among the latter, the unspecific toxicity of TMZ, poor solubility, and hydrolyzation are intrinsic characteristics, whereas the presence of the blood-brain barrier and some tumor properties, such as molecular and cellular heterogeneity and therapy resistance, have limited the therapeutic effects of TMZ in treating glioblastoma. Several reports have revealed that different strategies for TMZ encapsulation in nanocarriers overcome those limitations and have shown that they increase TMZ stability, half-life, biodistribution, and efficacy, offering the promise for future nanomedicine therapies in handling glioblastoma. In this review, we analyze the different nanomaterials used for the encapsulation of TMZ to improve its stability, blood half-life and efficacy, paying special attention to polymer- and lipid-based nanosystems. To improve TMZ drug resistance, present in up to 50% of patients, we detail TMZ combined therapeutic with i) other chemotherapies, ii) inhibitors, iii) nucleic acids, iv) photosensitizers and other nanomaterials for photodynamic therapy, photothermal therapy, and magnetic hyperthermia, v) immunotherapy, and vi) other less explored molecules. Moreover, we describe targeting strategies, such as passive targeting, active targeting to BBB endothelial cells, glioma cells, and glioma cancer stem cells, and local delivery, where TMZ has demonstrated an improved outcome. To finish our study, we include possible future research directions that could help decrease the time needed to move from bench to bedside.

## Current standard of care and treatment of glioblastoma

Glioblastoma (GBM), considered as grade 4 glioma attending to the 2021 CNS WHO classification 1, is the most common and lethal brain tumor in adults accounting for almost 50% of all cases. The annual incidence is around 1-5 per 100,000 population, and it presents a devastating prognosis with a median survival of 15-18 months and a five-year survival rate of less than 5% 2-4.

Surgery remains the best therapeutic option. Indeed, a greater extent of surgical resection is associated with better clinical outcomes. However, this is not always possible because usually the tumor affects areas in the eloquent cortex that are crucial for speech control, motor function, and the senses 5. Moreover, as GBM is a highly invasive tumor, radical resection of the tumor mass is not curative and the infiltrating tumor cells that remain within the surrounding area lead to later disease progression or recurrence 6. To enhance surgical resection, advances in the imaging techniques have been developed, including intraoperative MRI, diffusion tensor imaging, awake craniotomy, cortical mapping, stereotactic guidance, and fluorescent‑guided resection. For instance, studies have shown that fluorescent-guided resection enables patients to be progression-free for six months, by achieving more complete resections of contrast-enhancing tumors 7-9. However, even if maximal surgical resection remains important for the progression of patients, it seems that GBM is not completely cured with just a surgical answer 6. After surgery, the next step in the current standard treatment is the administration of radiation and chemotherapy. Indeed, the gold standard treatment for newly diagnosed patients consists of maximal surgical resection of the tumor (if possible), followed by the alkylating agent temozolomide (TMZ) in combination with 60 Gy of X-ray irradiation, fractionated in 30 sessions of 2 Gy each during six weeks, and six more cycles of TMZ for maintenance 10,11.

TMZ is an oral alkylating agent that transfers alkyl groups to guanine bases, causing DNA damage, which, if not repaired, causes apoptosis. This chemotherapeutic agent was added to the standard of care for GBM, when the clinical trial carried out in 2005 10 and additional trials 12,13 demonstrated that concurrent radiotherapy and TMZ followed by adjuvant TMZ significantly increased the median survival of the patients from 12.1 months (just radiotherapy) up to 14.6 months. In addition, studies revealed that the two-year survival rate was raised from 10.4% up to 26.5%.

Several omic studies 5,14-17 have revealed the existence of molecular, genetic, and epigenetic heterogeneity in GBM that have served as potential biomarkers with prognostic and diagnostic potential. These biomarkers include i) the expression of mutated isocitrate dehydrogenase (IDH) which is usually correlated with a better prognosis, ii) amplification of EGFR, iii) mutations in genes such as p53 and NF1, and iv) telomerase reverse transcriptase promoter mutations. Although this knowledge has served to better understand the disease, its translation into treatment efficacy has not yet been successful. In the case of TMZ, it has been described that TMZ response could depend on O6-methylguanine-DNA methyltransferase (MGMT) methylation status 10. MGMT is a DNA repair protein that removes alkyl groups from the O6 position of guanine in DNA. Methylation in MGMT causes its silencing and, thus, increases TMZ sensitivity, whereas no methylation in the MGMT promoter activates the gene expression, triggering enzyme repair and TMZ chemotherapy resistance 18-20.

**Figure 1:**
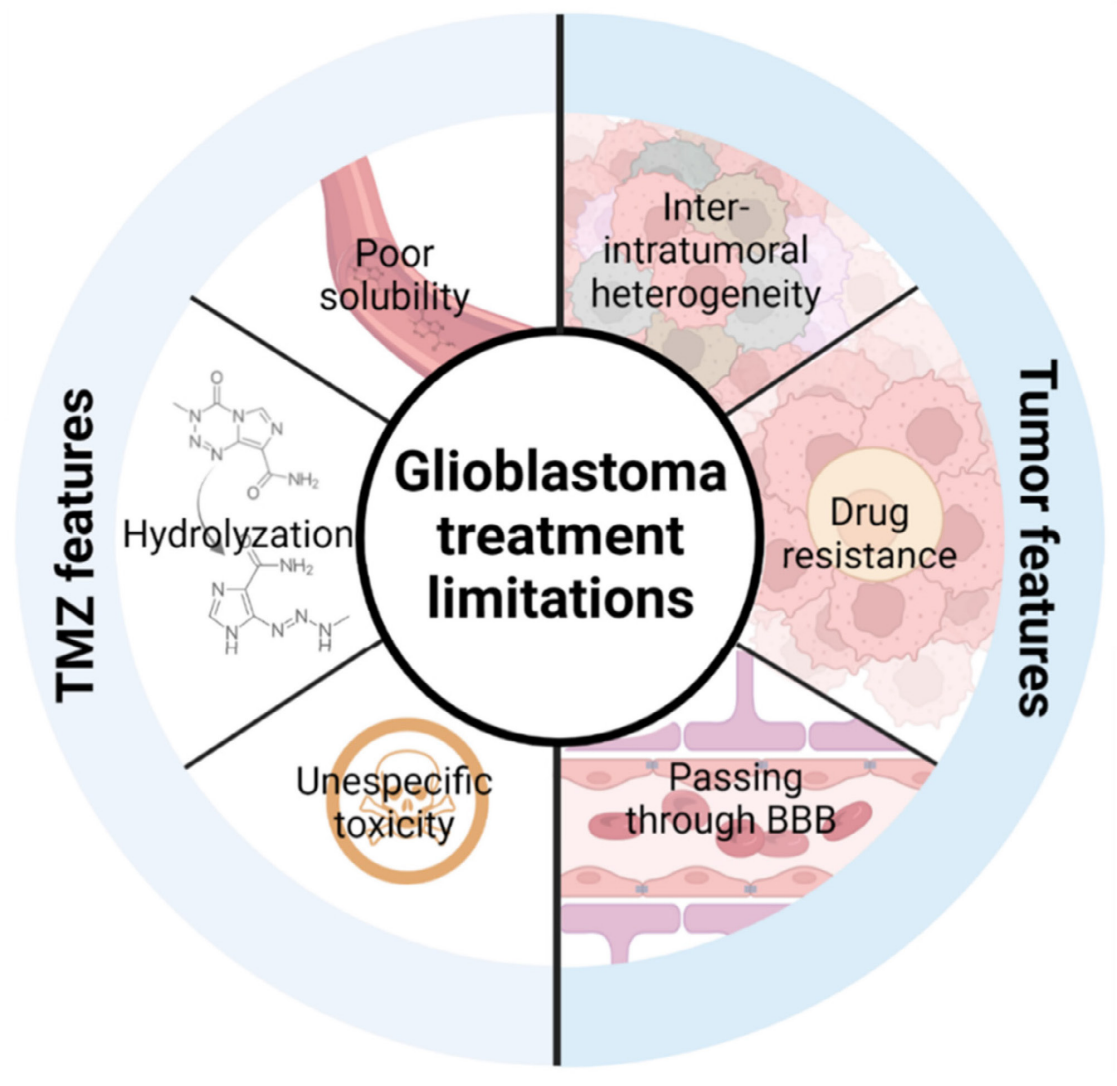
Limitations of current TMZ treatment that need to be overcome to improve its efficacy. TMZ features include its poor solubility, its hydrolyzation in contact with physiological medium and its unspecific toxicity. Tumor intrinsic features include inter- and intratumoral cell and molecular heterogeneity, drug resistance, and the blood-brain barrier.

Taking into account that TMZ is the only treatment that has demonstrated an advance in patient survival in clinical studies, strategies to improve its efficacy are promising to extend patient survival and increase their quality of life. In this review, we will describe the recent advances of TMZ encapsulation and how this strategy can improve **TMZ limitations** (its solubility, hydrolyzation and unspecific toxicity) and limitations due to the **intrinsic features of the tumor** including the blood-brain barrier (BBB), high inter- and intratumor cell and molecular heterogeneity and the development of therapy resistance 21,22 (**Figure 1**).

## Improving TMZ limitations

### TMZ mechanism of action

The metabolic pathway of TMZ is a pH-dependent reaction that begins when it comes into contact with a physiological medium. TMZ is hydrolyzed to 5(-3methyltriazen-1-yl) imidazole-4-carboxamide) (MTIC). This polar molecule is then degraded to AIC and the methyldiazonium cation. While AIC is secreted through the kidneys, the methyldiazonium cation is the responsible agent that transfers the methyl group to the DNA causing DNA damage followed by apoptosis when cellular repair mechanisms are unable to adjust the methylated bases 23.

MTIC has a short half-life (~ 2 h) and poor penetration through the BBB 24, so to achieve the desired therapeutic effect, patients receive high doses of TMZ for a long period, which can lead to serious systemic toxicity, drug resistance, and side effects 25,26. Nanomedicine can help overcome these problems: the encapsulation of TMZ offers different advantages including i) the improvement of TMZ's solubility and stability, ii) better TMZ brain accumulation, iii) reduction of high dosage administration, and subsequently, iv) a decrease of TMZ's side effects.

### Improving the solubility and stability of TMZ

**Figure 2:**
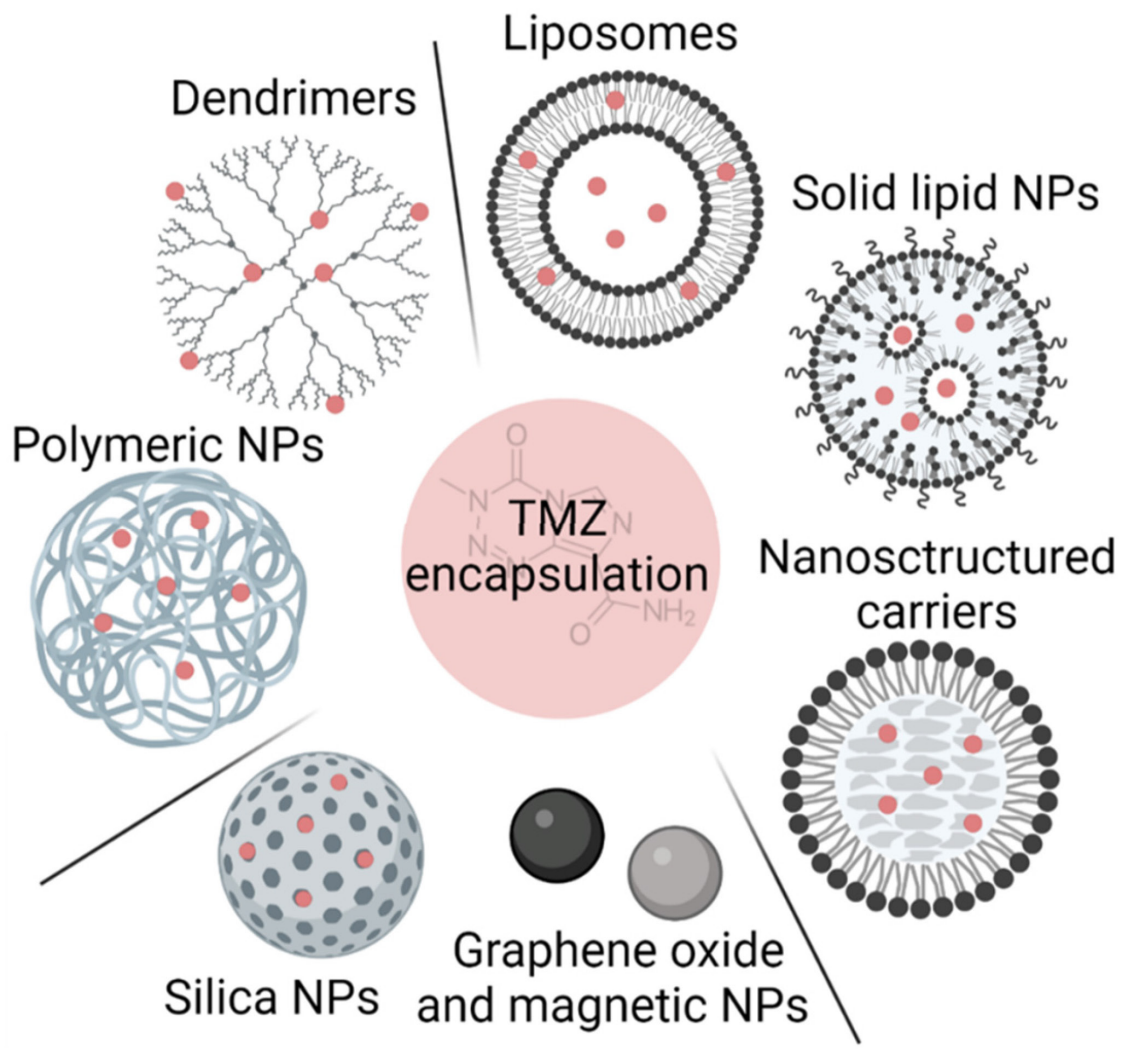
Summary of different possibilities for TMZ encapsulation

In this section, we analyze the current status of nanomaterials that have been used to encapsulate TMZ and improve its solubility and stability, and consequently its blood circulation time and efficacy. We detail the advantages and disadvantages of lipid- and polymer-based nanosystems and comment on newer systems based on other types of nanomaterials (summarized in **Figure 2** and **Table 1**).

#### Polymer-based nanosystems

Polymer-based nanosystems have been studied for biomedical applications due to their advantages, such as stability and long blood circulation time, significant water solubility or dispersibility, controlled size (10-200 nm), biodegradability, modifiable surface, and controlled drug release 27. Different nanosystems based on polymers have been used for TMZ encapsulation, among which polymeric nanoparticles (polymeric NPs) and dendrimers have been the most studied.

***Polymeric nanoparticles*** are solid, colloidal nano-sized (100-200 nm) particles that entrap or adsorb therapeutic agents into the polymer matrix or in the surface. The first polymeric NP for drug delivery to the brain was developed in 1995 28; however, it was not until 2011 that the first polymeric NP was described encapsulating TMZ 29. In that study, the researchers used polybutylcyanoacrylate nanoparticles, a minimally toxic, biocompatible, and biodegradable polymer with an easy synthesis and industrial scalable production 29. They tested and demonstrated for the first time in healthy Wistar rats that encapsulated TMZ was highly accumulated in the brain compared to free TMZ. Moreover, the study showed that TMZ's concentration was lower in the heart and kidney compared to free TMZ, suggesting that this system accumulated lower toxicity of TMZ in these organs 29. After this study, different researchers have performed additional approaches encapsulating TMZ in other polymeric NPs and analyzed the drug release profile *in vitro*. In particular, poly(lactic-co-glycolic acid) (PLGA) has been the most studied polymer. The first approach performed by Jain et al., described an enhanced release pattern of TMZ from PLGA nanoparticles. Free TMZ was dissolved in ~2h, while encapsulated TMZ had a sustained release pattern up to 120h, thus increasing TMZ availability 60 times 30. Additional reports have shown that the sustained release pattern could be from over 3 days 31 up to 9 32 and even 10 days 33.

Although PLGA nanoparticles increase the stability of TMZ in physiological media, their loading capacity and encapsulation efficacy (EE) are usually poor at just 2-4% and 17-50%, respectively. To overcome these disadvantages, NPs composed of different polymers have been studied. For example, PLGA and chitosan nanoparticles achieved an encapsulation efficiency of 27.3% 34 and PLGA nanoparticles with polyethylene glycol (PEG) increased their EE to 64.5% 35. Moreover, nanoparticles based on polymers such as chitosan 36-38, polysaccharides 39, PLA 40 or albumin 41,42 have confirmed the same release pattern, thus improving TMZ stability.

In addition to polymeric NPs, other polymeric structures such as dendrimers are being studied for TMZ encapsulation*.** Dendrimers*** are branched polymeric molecules that consist of an internal core and repeated external branching units (**Figure 2**). Their structure allows i) conjugation of active drugs or functional groups in the peripheral branching framework by covalent binding, hydrogen bonding, or electrostatic adsorption and ii) entrapping drugs between the segmented cavities of the polymer blocks. Sharma et al. developed a PAMAM-chitosan conjugate and tested them *in vitro* in U251 and T98G cell lines showing that dendrimers caused higher cell death in both cell lines 43. They also performed pharmacokinetic analysis *in vivo* and demonstrated that the formulation had a sustained release with a TMZ half-life one and a half times higher, increasing TMZ brain concentration by almost twofold when it was administrated encapsulated 43.

In general, polymer-based nanosystems have demonstrated the ability to increase the stability of TMZ and control its release, which could be translated into an increase in the blood circulation time *in vivo*, and hence, an improvement in TMZ brain accumulation.

#### Lipid-based nanosystems

Lipid-based nanosystems have been widely studied for TMZ encapsulation. They have different advantages, including i) encapsulation of both hydrophobic and hydrophilic molecules, ii) improvement of drug solubility and blood half-life time, iii) low toxicity and safe biodegradation, and iv) the possibility of controlled drug release. The most characterized nanosystems for TMZ encapsulation in GBM are liposomes and nanostructured carriers (NSCs) (**Table 1**).

***Liposomes*** are small spherical vesicles composed of a phospholipid bilayer in an aqueous medium, with a structure and composition very similar to the phospholipids of the cell membrane; thus, they are nontoxic and biodegradable. The first articles reporting TMZ encapsulation in liposomes were carried out in 2009 44 and 2010 45. However, the first study reporting a liposomal formulation encapsulating TMZ for GBM treatment was in 2015 46. In this initial approach, the authors studied the pharmacokinetics and biodistribution of administrated liposomes in healthy rabbits and mice, showing that liposomal encapsulation of TMZ prolonged its half-life in plasma. Moreover, TMZ brain concentration was higher in animals administrated with the liposomal formulation. They also observed that less liposomal TMZ accumulated in the heart and lungs, which could indicate fewer TMZ side effects in these tissues. However, the liposomal TMZ concentration was higher in liver, kidney, and spleen, highlighting a note of caution regarding this strategy. In this case, the high level of TMZ in the liver and spleen could be explained by the fact that macrophages in these organs tend to take up nanoparticles. Plasma proteins bind to the surface of nanoparticles and are recognized by the reticuloendothelial system, clearing them from the liver and spleen 47.

Therefore, although the blood circulation time was increased compared to that of the free drug, it was still low due to their rapid elimination by macrophages from the reticuloendothelial system. To solve this problem, different researchers have incorporated PEG on the surface of liposomes, by a process called PEGylation 47. This polymer is hydrophilic and inert and provides a steric barrier on the surface of liposomes, minimizing plasma protein binding and enhancing nanoformulation stability. A study performed by Hu et al. 48 not only demonstrated that PEGylated liposomes increased the plasma concentration of TMZ and were more concentrated in the brain, but they were also able to delay the liposomes' clearance, suggesting that PEGylation is a good strategy for overcoming the reticuloendothelial system.

However, it has been reported that repeated intravenous injections of PEGylated liposomes led to a phenomenon called “accelerated blood clearance,” where the induction of anti-PEG antibodies in the first injection triggers the removal of the formulation from the body 49,50. Thus, ***solid lipid nanoparticles*** (SLNPs) were designed to improve the stability of nanoformulations. SLNPs are a new generation of colloidal lipid carriers composed of physiological lipids that are in the solid phase at room and at physiological temperatures 51,52. Huang et al. demonstrated that, similar to liposomes, the release pattern of TMZ from this nanosystem was slow, increasing the plasma concentration of TMZ and brain accumulation from 6.7% up tp 13.25% 53.

SLNPs present some advantages over liposomes such as better stability and scalable industrial production. However, they still have some drawbacks to overcome. They present a moderate drug-loading capacity and can expulse the load due to the crystallization process under storage conditions 52. For this reason, nanostructured carriers are being developed. ***Nanostructured carriers*** are a second generation of lipid-based nanosystems that combine in their structure solid and liquid lipids at room and at physiological temperatures. The use of both types of lipids improves the encapsulation capacity and avoids drug expulsion 52. In 2016, Qu et al. published an article where they compared the best nanosystems to carry and deliver TMZ between NSCs, SLNPs, and polymeric NPs 54. They concluded that NSCs had the best anti-tumor activity *in vitro* and *in vivo*. Although all nanoformulations showed significantly higher efficacy than the free drug, the IC_50_ value of NSCs was 4 and 7 times lower than those of SLNPs and polymeric nanoparticles, respectively. Moreover, U87 solid tumors in mice were inhibited by 85% in the case of NSCs, while it was by 59% and by 45% in the cases of SLNPs and polymeric NPs, respectively. These differences might be attributable to the better capacity of NSCs to enter the tumor and release the drug into cancer cells. Moreover, when comparing the efficacy of TMZ loaded in SLNPs with that of NSCs, Wu et al. demonstrated that NSCs had a significantly better tumor inhibition efficiency both *in vitro* and *in vivo* 55. While TMZ loaded NSCs displayed a tumor inhibition rate of 70% compared with control animals, SLNPs had a tumor inhibition rate of 43%.

#### Other nanomaterials

Lipid-based and polymeric-based nanosystems have been widely studied for the encapsulation of TMZ for GBM. Indeed, clinical studies examining the efficacy of other drugs, such as doxorubicin or irinotecan, encapsulated in liposomal formulations suggest that these nanosystems may still be a feasible approach against GBM in the near future. However, different nanomaterials are also being studied to develop new strategies, including mesoporous silica nanoparticles, graphene oxide nanoparticles, and magnetic nanoparticles (**Table 1**).

Mesoporous silica nanoparticles are silicon oxide porous structures that exhibit good biocompatibility and nontoxicity 56,57. Preliminary studies *in vitro* have demonstrated that TMZ is efficiently encapsulated in silica nanoparticles, having a better antitumoral effect than free TMZ 58-60. However, more studies are needed in this new field, especially with *in vivo* models.

In addition to silica nanoparticles, other nanomaterials have been studied for the delivery of TMZ, among which, there has been one study reported that used graphene oxide nanoparticles. It was demonstrated that this strategy increased cell inhibition in rat glioma cells 61. In addition, magnetic nanoparticles are another nanomaterial that has been used for the delivery of TMZ. Indeed, Dilnawaz et al. demonstrated in T98G cell line 2D cultures and 3D spheroids that TMZ loaded in these nanoparticles triggered cell death induction 62.

#### Combinations of nanomaterials

Different studies have proposed that the combination of different nanomaterials, such as silica nanoparticles with polymers, improves the nanoformulation in different diseases including COVID-19 and breast cancer 63-65. Moreover, Mazarei et al. 66 designed TMZ-loaded selenium nanoparticles functionalized with chitosan and Eudragit®, a pH-dependent polymer that is dissolved in a medium with pH > 5.5, to enhance site-specific drug delivery. They measured the effect of their nanoformulation *in vitro*, demonstrating that i) the stability of TMZ improved at acidic and neutral pH by reducing the IC_50_, ii) increased the cellular uptake, and iii) treated cells decreased the expression of important genes such as MGMT and induced more efficiently apoptosis with higher cytotoxicity.

Furthermore, other nanomaterials such as superparamagnetic nanoparticles 67-71 have been used to design TMZ-loading nanosystems to add new functionalities 51. The use of superparamagnetic nanoparticles offers various advantages, including the possibility of i) actively targeting them under an external magnetic field, ii) gaining visibility in magnetic resonance imaging (MRI) by reducing both T1 and T2/T2* relaxation times, and iii) magnetic hyperthermia (discussed in the Section "TMZ with photodynamic therapy, photothermal therapy, and magnetic hyperthermia). Hence, adding superparamagnetic nanoparticles to the nanosystems might offer these advantages. For instance, Ling et al. combined superparamagnetic nanoparticles with PLGA and Tween 80 for TMZ encapsulation 67. In addition to an excellent sustained release profile and an inhibitory cell proliferation effect *in vitro*, this study demonstrated that the combination of superparamagnetic nanoparticles with polymeric nanoparticles could be used as contrast agents. Bernal et al. 68 tested their nanosystem composed of polymeric (PEG-PLA-PCL) superparamagnetic-bearing nanoparticles *in vitro* and *in vivo* with local delivery (convection-enhanced delivery, CED). They showed by MRI, due to the magnetic nanoparticles, that CED could effectively distribute their nanosystem with just some local edema but with no parenchymal changes. Moreover, glioma cells trapped the nanosystem and the TMZ release prolonged the survival of the animals compared to the control ones. More recently, the same group demonstrated similar results in an *in vivo* dog model 69, showing that the encapsulation of superparamagnetic nanoparticles adds the advantage of image guidance.

### Improving TMZ resistance: Combined therapy

**Figure 3:**
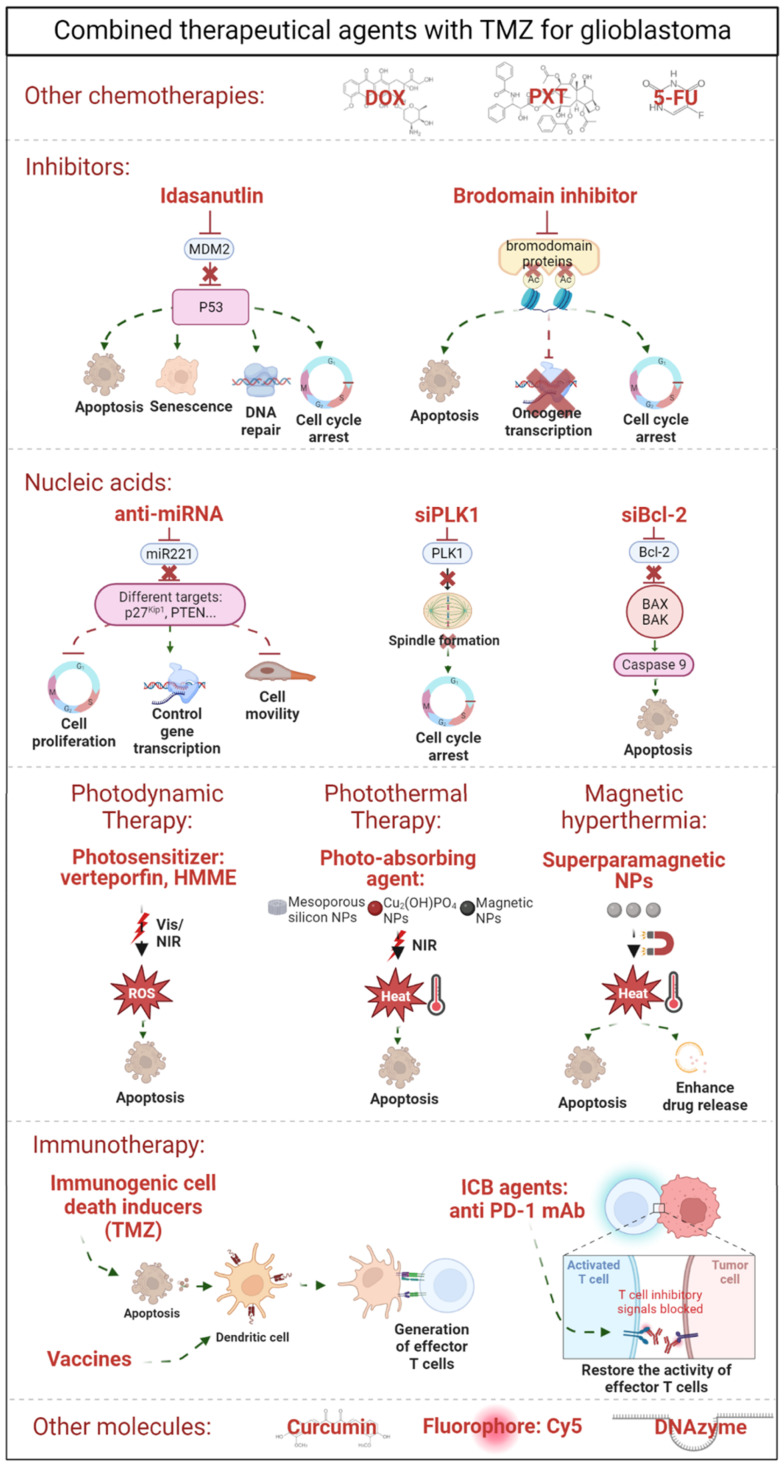
Schematic illustration of combined therapeutical approaches with TMZ for GBM treatment, including i) other chemotherapies, ii) inhibitors, iii) nucleic acids, iv) alternative treatments, such as photodynamic therapy, photothermal therapy, and magnetic hyperthermia, v) immunotherapy, and vi) other less-explored molecules.

The encapsulation of TMZ in nanosystems improves the stability and brain accumulation of the drug compared with free TMZ administration. However, it has been reported that up to 50% of patients do not respond favorably to TMZ therapy. As other studies have demonstrated that the combination of therapies achieved a synergic effect between them 72,73, researchers are studying the co-delivery of TMZ with other therapeutic agents. In fact, nanosystems are being developed to be encapsulated together with TMZ. These agents include other chemotherapeutic agents (paclitaxel 74, doxorubicin 37, and 5-Fluoracil 39); inhibitors 75,76; nucleic acids 58,77-80; photosensitizers for photodynamic therapy 81,82; other nanomaterials for photothermal therapy 83,84 and magnetic hyperthermia 85-87 and other molecules 59,62,88 (summarized in **Table 2** and **Figure 3**).

#### TMZ with other chemotherapies

Several studies have reported that polymer-based nanosystems could be used to co-encapsulate TMZ with other chemotherapies including doxorubicin 37, paclitaxel 74 and 5-Fluoracil 39. In studies carried out by Di Martino 37,39, both doxorubicin and 5-Fluoracil were co-encapsulated with TMZ in polymeric NPs. Behrooz et al., on the other hand, used dendrimers composed of PAMAM to deliver TMZ with paclitaxel to U87 CD133+ and CD44+ stem cells 74. They demonstrated that this nanosystem was able to increase the early apoptosis from 28.2% in the case of the administration of the free drug, up to 73.3% with the encapsulated co-delivery. In addition to polymer-based nanosystems, studies reporting the co-encapsulation of TMZ with other chemotherapies, such as vincristine in SLNPs and NSCs, suggesting that lipid-based nanosystems could also be used for the co-delivery of TMZ with other chemotherapies 55.

#### TMZ with inhibitors

To try diverse therapeutical strategies, researchers have encapsulated TMZ with specific inhibitors. For instance, polymeric NPs, such as PLGA and poly(styrene-b-ethylene oxide) (PS-b-PEO) nanoparticles have been used to encapsulate TMZ together with an inhibitor of the oncoprotein MDM2, called idasanutlin 75. Idasanutlin has been described as a MDM2 inhibitor of the nutlin class that binds specifically to MDM2 with > 100-fold selectivity for GBM in various cell lines 89. The administration of both therapies was performed in glioma stem cells (GSCs) and they proved that both treatments had a synergic effect increasing the cytotoxicity percentage from ~ 10% in the case of TMZ-loaded NP administration compared to ~ 75% when they were exposed to polymeric NPs with both therapies 75. Moreover, PEGylated liposomes were studied for the co-delivery of TMZ with a brodomain inhibitor 76, a molecule that binds competitively to acetyl-lysine binding motifs, triggering DNA damage and apoptosis 90. They showed that both treatments were more efficient together than the administration of TMZ and the inhibitor separately in U87 and GL261 tumor-bearing mice.

#### TMZ with nucleic acids

Nucleic acid-based therapies have emerged as a powerful strategy for the treatment of brain tumors due to their direct, effective, and lasting therapeutic effect 91. However, due to their instability, their difficulty traversing biological barriers and their off-target effect, they are perfect candidates to be encapsulated together with TMZ.

As a proof of concept, Chen et al. co-loaded TMZ with an enhanced green fluorescent protein gene in NSCs, confirming that NSCs could efficiently achieve stable gene and drug delivery, reduce the tumor growth in mice, and extend their overall survival due to the effect of TMZ 80. In addition to NSCs, polymer-based structures, such as polymeric micelles, have been shown to be effective in the co-delivery of TMZ with nucleic acids. Polymeric micelles (10-200 nm) are colloidal systems composed of polymeric molecules dispersed in liquid. They have a core-shell structure, with an inner core composed of a hydrophobic region, and a corona composed of a hydrophilic part for the micelle stabilization 27. Shi et al. co-delivered TMZ with a *polo*-like kinase-1 siRNA (PLK-1) 78, which is involved in the spindle formation and chromosome segregation during mitosis and is overexpressed in glioma tissues and whose inhibition causes cell cycle arrest and cell apoptosis 92. Shi et al. demonstrated this not only in cell cultures, but also in a U87 orthotopic mouse model. They showed that, in addition to the tumor size reduction, the median survival of the mice and their weight were 1.2 and 1.17 times higher, respectively, compared to animals administrated with only TMZ 78. In addition, Peng et al. encapsulated TMZ and a Bcl-2 siRNA that promotes cell apoptosis in a triblock copolymer micelle using poly(ε-caprolactone) (PCL), poly(ethylenimine) (PEI), and PEG 79. The three polymers self-assembled via hydrophobic interaction encapsulating the drug in the hydrophobic core. Subsequently, the siRNA was complexed with the micelles through electrostatic interaction. They demonstrated that in rats bearing an orthotopic glioma model, micelles containing both strategies reduced the tumors from ~ 200 mm^3^ and ~ 170 mm^3^ in the case of the administration of siRNA and TMZ, respectively, to ~ 75 mm^3^ with both therapies 79. Moreover, in a recent study, Wang et al. co-delivered TMZ with an EGFR-siRNA in polymeric nanoparticles and observed that while in mice treated with TMZ, tumors grew rapidly with a glioma inhibition rate of 164.3, the tumor inhibition rate for animals treated with both drugs was 15.3, suggesting a high antitumor efficacy and synergic effect 77.

The work carried out by Bertucci et al. combined TMZ with an anti-microRNA in silica nanoparticles. Various studies have reported that the abnormal expression profile of miRNAs is correlated with the pathogenesis of cancer cells, tumor progression, and drug resistance 93. Indeed, in glioma, one of the upregulated miRNAs is the miR221 94, which is responsible for the regulation of several key target genes of cell-cycle progression 95. Interestingly, different reports have also shown that the downregulation of miR221 sensitized glioma cells to temozolomide 96,97. Thus, Bertucci et al. co-delivered an anti-mR221 and TMZ *in vitro* and demonstrated that their nanosystem induced apoptosis by combining a synergic effect of both strategies 58.

#### TMZ with photodynamic therapy, photothermal therapy, and magnetic hyperthermia

In addition to chemotherapy, inhibitors, and nucleic acids, researchers are studying other therapeutic approaches, including photodynamic therapy (PDT), photothermal therapy (PTT), and magnetic hyperthermia.

Photodynamic therapy consists of the use of a photosensitizer (PS), which after light stimulation (usually with visible or NIR light), forms highly reactive oxygen species that interact with cellular structures and lead to cell damage and possible cell death (**Figure 3**). The FDA recently approved 5-aminolevulinic acid (ALA) for fluorescence-guided surgery on high-grade gliomas 7,98. Indeed, ALA and its derivatives are one of the most studied compounds as PSs for PDT in GBM. Although studies have proved the beneficial effect of PDT in GBM (summarized in 99,100), in this review, we are focusing on works that have studied the synergic effect of TMZ with PDT. To our knowledge, the first article reporting combined therapy of TMZ with PDT was performed by Zhang et al. 101, who administrated both treatments (hematoporphyrin monomethyl ether, or HMME, as a second-generation PS and TMZ) in glioma-bearing rats, demonstrating that their combination increased glioma cell apoptosis and also prolonged the overall survival of rats from approximately 21 days, 37 days, and 33 days in control rats and rats treated with PDT or TMZ alone, respectively, up to 51.55 days in the case of PDT-TMZ treated animals. A more recent study has demonstrated that the use of HMME as a PS for PDT-TMZ combined therapy inhibits cell migration and invasion, and more specifically induces mitochondrial-associated apoptosis 102. Most second-generation PSs are lipophilic with a lack of solubility in aqueous media; thus, their encapsulation in nanocarriers has been proposed to avoid their aggregation and improve their solubility in aqueous media 103. For example, in a study performed by Pellosi et al. polymeric micelles were used for the co-delivery of TMZ in combination with verteporfin, an FDA-approved PS 81. Micelles were prepared with Pluronic®, a tri-block copolymer made of PEO, poly(propylene oxide) (PPO) and PEO, where the Pluronic PEO block is hydrophilic and water soluble and PPO is hydrophobic and water insoluble. They showed the synergic response between the two therapies in three different cell lines (U87, T98G, U343), including a TMZ-resistant cell line 81.

Photothermal therapy is a noninvasive therapy that requires the use of an external near-infrared laser to irradiate the tumor and a photo-absorbing agent that will convert the light energy into heat, triggering localized hyperthermia. Cancer cells are more sensitive to temperature than normal cells 104. Thus, increasing therapeutic temperature (42-47 °C) in the tumor region by photothermal therapy might be a feasible strategy for irreversible cellular damage and cellular apoptosis (**Figure 3**). Different nanomaterials have been used as photo-absorbing agents for GBM, including Cu_2_(OH)PO_4_ NPs 105, porous silicon NPs 83, and iron oxide NPs 82. Chen et al. demonstrated that under NIR irradiation, Cu_2_(OH)PO_4_ nanoparticles increased the temperature of the tumor region, which consequently diminished the tumors even completely 105. As the next step, Zeng et al. 83 combined PTT with TMZ using porous silicon NPs. In fact, it has been reported that heating the tumors at 40-42 °C increases the blood flow and the partial pressure of oxygen, enhancing the delivery of chemotherapies to tumor cells 106. Hence, this study showed that while the inhibition rates of plain NPs, TMZ, irradiated NPs, and encapsulated TMZ were 16.5%, 37.2%, 24.2%, and 57.2%, respectively, the combination of encapsulated TMZ plus PTT increased the rate up to 71.5% 83. In addition to porous silicon NPs, iron oxide NPs have also been studied for TMZ-PTT treatments 82. Kwon et al. loaded in Fe_3_O_4_ NPs TMZ together with indocyanine green, a photothermal agent and photodynamic photosensitizer that under NIR laser irradiation can convert absorbed NIR light to thermal energy and reactive oxygen species (ROS). Indeed, Fe_3_O_4_ magnetic NPs are known to have good photothermal conversion efficacy in the NIR region and low toxicity. Hence, this study has merged chemo-photothermal-photodynamic therapy and demonstrated synergic U87 cytotoxicity, inducing ROS generation and cell death 82. In a more recent study, Cao et al. combined hollow mesoporous copper sulfide nanoparticles with TMZ for PDT and mild PTT 84. Under a tumor acidic microenvironment and NIR irradiation, copper NPs dissociated Cu^2+^ to consume glutathione by redox reaction, generating Cu^+^ that converts H_2_O_2_ into highly toxic OH for PDT. Moreover, the mild photothermal effect produced (rise of temperature to 40-44 °C), increased blood flow and, hence, TMZ delivery, showing a decrease of the relative tumor volume (V/V_0_) from almost 5 to almost 0 84.

Magnetic hyperthermia is another therapeutic strategy that has been combined with TMZ in GBM. Actually, superparamagnetic nanoparticles heat up (preferably to 42-45 °C) under an external alternating magnetic field, which causes changes in the functions of the cell membrane, proteins, and the synthesis of nucleic acids, triggering apoptosis (**Figure 3**) 107. Indeed, Minaei et al. designed polymeric nanoparticles containing both superparamagnetic nanoparticles and TMZ. They showed *in vitro* in C6 cancer cells that localized heating-triggered TMZ release from nanoparticles resulted in a synchronized effect of both chemotherapy and hyperthermia 85. In addition to polymer-based nanosystems, Yao et al. encapsulated TMZ and ferroferric oxide (Fe_3_O_4_) in temperature-sensitive liposomes to enhance the drug release and combine both TMZ and magnetic hyperthermia effects *in vitro* 87. Marino et al. embedded magnetic NPs and TMZ in SLNPs and showed in U87 spheroids that both treatments, hyperthermia and TMZ, were able to decrease their size from ~ 550 µm in control spheroids to ~ 150 µm 86, demonstrating the possible use of lipid-based nanosystems for magnetic NPs loading systems.

#### TMZ as immunogenic cell-death (ICD) inducer and immunotherapy

Immunotherapy is based on re-educating and harnessing the patient's immune response against tumors. This method includes immune checkpoint blockade (ICB) agents, therapeutic vaccines, adoptive cell therapy, monoclonal antibodies (mAbs), and oncolytic viruses 108. Although ICB agents have demonstrated success in patients with melanomas and other tumors 109,110, most patients with solid tumors do not respond to immunotherapy. Indeed, less than 15% of cancer patients do respond to ICB agents 111. In the case of glioblastoma patients, results from phase III clinical trials with ICB agents and vaccine therapies have not demonstrated a major benefit in terms of patient survival or immune modulation 112. However, two recent studies have raised hope for the immunotherapy in GBM: in fact, the administration of PD-1 mAbs prior to tumor resection increased local and systemic antitumor immune responses 113. Moreover, a phase II clinical trial has shown that patients' survival increased after the administration of a combination of different immunotherapies 114. Thus, it seems that stimulating the immune system at different times and locations or combining it with other treatments could be possible and effective. It is thought that one possibility to increase the immune response against GBM is by stimulating antigen release from dying tumor cells and their presentation to dendritic cells by immunogenic cell-death (ICD) inducers, such as radiotherapy or TMZ (**Figure 3**) 115. It has been demonstrated that modifying the dose (metronomic doses) 116 or the administration route (local delivery) 117 of TMZ together with ICB agents has increased the efficacy of anti-PD-1 antitumor efficacy and vaccine therapies 118.

It is true that the administration of TMZ in all these examples was done freely, and, as it has been shown that the route of administration can play a key role in improving the efficacy of immunotherapies, the use of drug delivery systems could improve the penetration of TMZ and, hence, its role as an ICD inducer. In fact, there are examples with other chemotherapies such as DOX 119 and MIT 120 encapsulated in polymersomes and hydrogels, respectively, that have been used as ICD inducers together with radiotherapy and a siRNA against an immunosuppressive mediator, respectively, showing promising results *in vivo* in glioblastoma.

#### TMZ with other molecules

In addition to all the approaches featuring co-delivery of TMZ with different therapeutic choices, other less-explored molecules are also being studied, including curcumin, dyes, and DNAzyme.

Different nanomaterials, such as magnetic nanoparticles 62 and NSCs 88, have been used for the co-delivery of TMZ with curcumin, a natural compound that has demonstrated anticancer activity inducing differentiation, apoptosis, and inhibition of cell growth 121. The nanosystem proposed by Xu et al. 88 exhibited enhanced inhibitory effects on glioma cells due to a combination of S-phase cell-cycle arrest and induced apoptosis. They also tested the formulation in a C6-bearing mouse model and concluded that, while drugs accumulated better in the brain and in tumor sites when they were encapsulated, the co-delivery performed a significant synergic effect between TMZ and curcumin, decreasing the tumor weight from ~ 0.8 and 0.75 g in animals with just curcumin or TMZ administration, respectively, to < 0.5 g with both treatments 88.

A recent study carried out in 2020 by Nie et al. confirmed that silica nanoparticles could be used for co-delivery of TMZ with a DNAzyme (for the gene silencing of MGMT). They were able to cleavage MGMT mRNA, knock down the MGMT protein, and improve the therapeutic effect of TMZ *in vitro* in T98G glioma cells 59.

Dual encapsulation of TMZ with other therapeutic agents seems to be a good strategy to increase the sensitivity of cells to TMZ and improve the antitumoral effect of both molecules. In addition to the co-delivery of two therapeutic agents, novel approaches have been developed to extend the use of nanosystems. Indeed, Schmitt et al., co-encapsulated TMZ with a dye (Cy5) as a potential diagnostic probe in chitosan nanoparticles. Moreover, they showed that the co-encapsulation of the Cy5 dye could provide a marker for real-time monitoring of the delivery and accumulation of the nanoparticles while showing TMZ efficacy 34.

## Improving GBM limitations

As mentioned in the previous section, the encapsulation of TMZ not only improves its stability and protection but also enables the possibility of dual encapsulation with other therapeutic strategies, achieving a synergic effect. In addition to these advantages, drug encapsulation can improve the delivery of the treatment into tumoral tissue and cells via passive targeting, active targeting, or local delivery.

### Passive targeting

Tumor vessels differ from normal vessels in several ways. Most tumor vessels have an irregular diameter and an abnormal branching pattern, making it difficult to classify them as arterioles, capillaries, or veins 122,123. Moreover, they do not have a smooth muscle coating, and large vessels usually have thin walls. Besides, the same vessels have an incomplete basement membrane, an abnormal pericyte coat and are unusually leaky 124,125. This leakiness can lead to extravasation of proteins and might facilitate the circulation into the blood stream of tumor cells—thus forming metastases—as well as other molecules such as radioisotopes 124,126,127. Indeed, it is thought that this leakiness is due to a defective endothelial monolayer, which has spaces or pores between poorly connected, branched lining cells 123.

It has been shown that the pore size between new tumor blood vessels can range from 10 up to 2000 nm 123,128. In contrast, blood vessels in healthy tissues have a pore size of approximately 10 nm. Consequently, when a drug is systemically administrated, while free chemotherapeutic molecules (< 1 nm) reach not only tumors but also normal cells, nanocarriers (~ 100-200 nm) may preferentially pass through the pores of tumor vessels 129.

**Figure 4:**
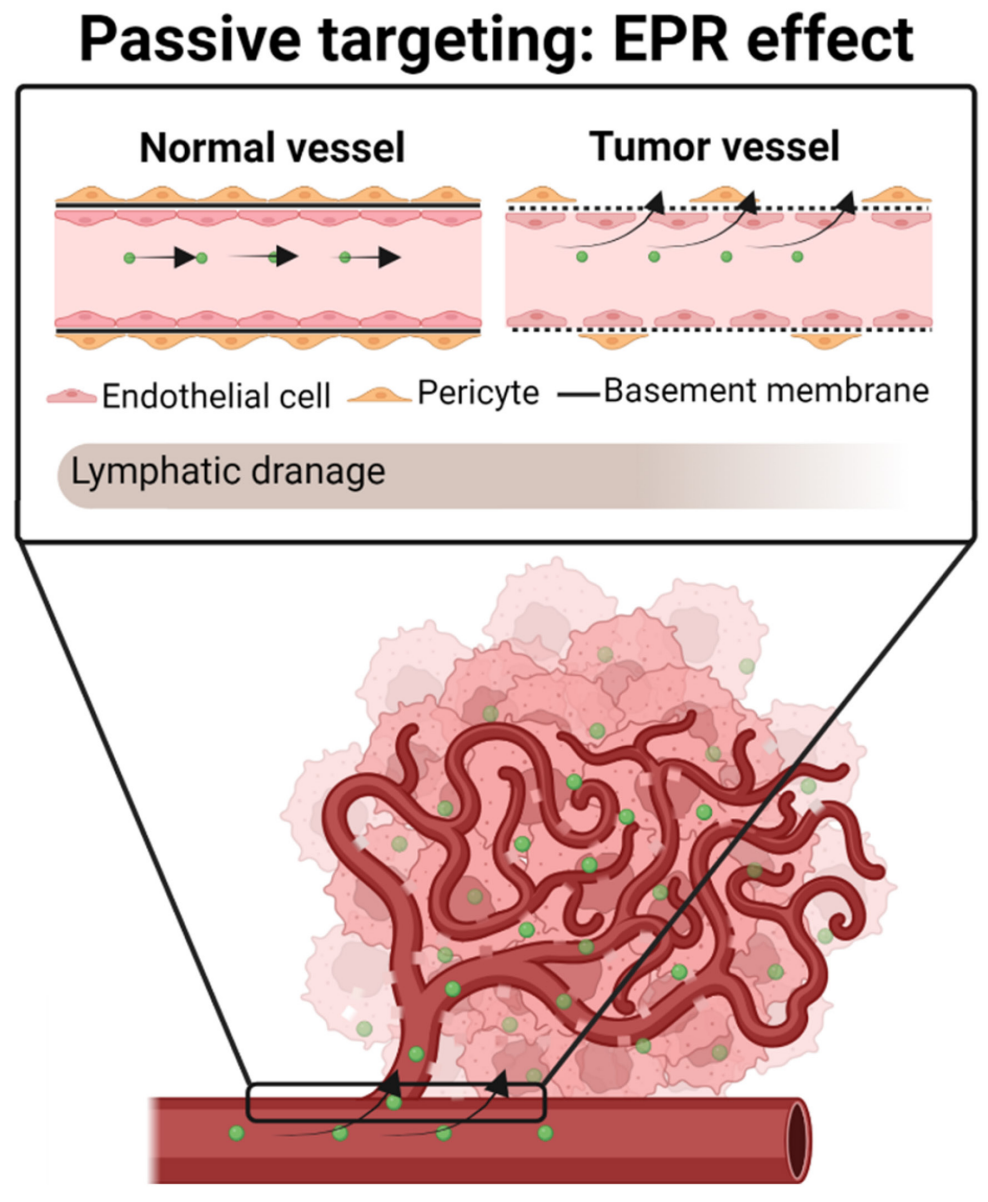
Illustration of local delivery for GBM including implantable wafers, convection-enhanced delivery and hydrogels, where implantable or *in situ*-formed hydrogels can be distinguished.

Matsumura and Maeda were the first to demonstrate that nanoparticles can extravasate through leaky blood vessels to reach the tumor space and be retained there, due to the poor lymphatic drainage of tumors 130. This phenomenon is known as the enhanced permeability and retention effect (EPR effect) (**Figure 4**). It is based on several pathophysiological characteristics of solid tumors, such as i) the massive irregular neovascularization of tumors, ii) the elevated expression of inflammatory factors that sustain the EPR effect, including prostaglandins, VEGF, interleukin 2 and 6, and iii) inefficient lymphatic drainage, which retains the extravasated nanosystems 130-132.

In the case of TMZ-loaded nanosystems, studies have demonstrated that its encapsulation in lipid-based nanosystems, including liposomes, PEGylated liposomes, SLNPs, and NSCs 46,53,55,88, have enhanced the brain accumulation of the TMZ *in vivo*, increasing the therapeutic effect of TMZ and reducing its toxicity, compared with the administration of the free drug.

### Active targeting

GBM neovasculature is highly permeable and the EPR effect has been demonstrated *in vivo*. However, disruption of the BBB remains a local event that is more evident in the tumor core but absent in the growing margins. In conclusion, it has been reported that with the ERP effect there is an accumulation of therapeutic agents in necrotic tumor areas with an almost undetectable concentration of drug in the peritumoral regions 133,134. Therefore, other strategies capable of optimizing the BBB crossing and the specific interaction between TMZ and targeted cells, including endothelial cells, glioma cancer cells, and glioma stem cells, are needed.

**Figure 5:**
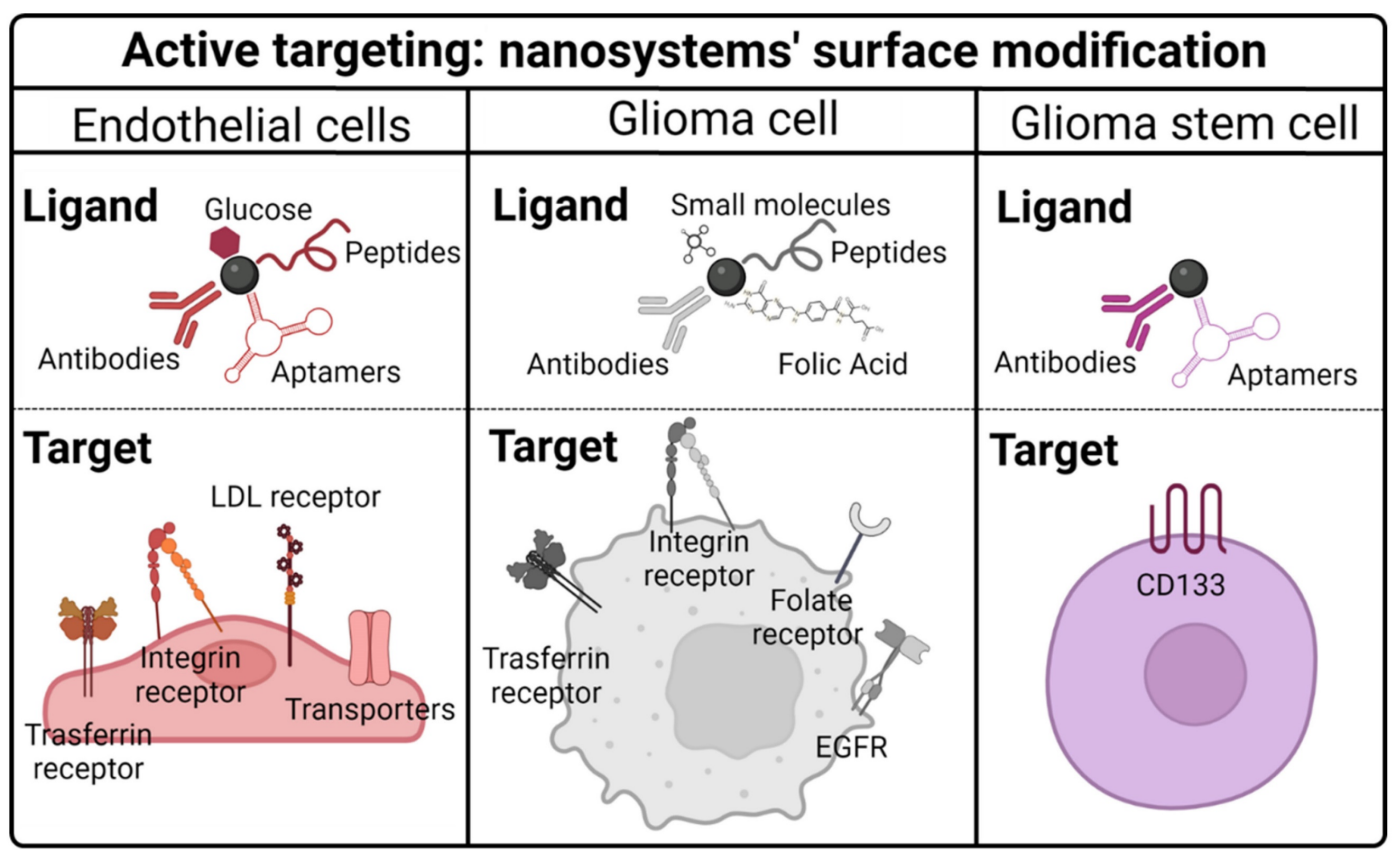
Schematic illustration of the most ligands and targets used in TMZ encapsulation systems.

Modification of nanosystem surfaces with ligands could be a possible strategy for this active targeting. Indeed, nanomaterials have a high surface/volume ratio and a highly reactive surface; thus, they have been functionalized with different ligands to target TMZ to different cell types 20 (**Table 3, Figure 5**).

#### Targeting BBB endothelial cells

It has been described that ***transferrin*** (Tf), ***integrin***, and ***low-density lipoprotein*** (LDL) receptors are overexpressed in BBB endothelial cells. To our knowledge, the first approach using ***transferrin*** receptor as a target molecule was reported in 2015 135, where researchers designed a TMZ-loading liposome functionalized with an anti-Tf receptor antibody. First, they demonstrated *in vitro* in U87 and U251 and in two TMZ-resistant cell lines (U87R and T98G) that encapsulation of TMZ improved the antitumor activity of TMZ in both TMZ-sensitive and TMZ-resistant cell lines 135. In addition, they analyzed the *in vivo* efficacy in intracranial U87 and subcutaneous T98G tumors. Systemic administration of the liposomal formulation triggered a substantial inhibition of tumor growth, as well as an increase in cell apoptosis and animal survival compared with animals with free TMZ administration. Moreover, they demonstrated a decrease in the toxic effects of encapsulated TMZ 135. A design composed of PEGylated liposomes with a Tf receptor-targeting moiety was also studied to co-deliver TMZ and a brodomain inhibitor 76. Mice bearing U87 and GL261 intracranial orthotopic tumors presented more prolonged survival, smaller tumor size, and lessened toxic effect when they were administrated with both encapsulated treatments 76. In addition to liposomes, SLNPs have been modified to target a Tf receptor. A study carried out by Jain et al. showed that Tf-TMZ-SLNPs were accumulated more in the brain of rats compared to unconjugated SLNPs and control animals (free TMZ) 136. Besides, they revealed a 70% lower hemolytic toxicity in Tf-TMZ-SLNPs animals, indicating good biocompatibility of the system. Furthermore, Tf-TMZ-SLNPs had a better tumor-inhibitory effect than free TMZ or plain SLNPs. Similar results in terms of active targeting were also achieved when Fu et al. targeted the Tf receptor by using an aptamer 137, a short single strand of DNA or RNA that has a specific 3D structure capable of binding with high affinity to proteins 148. They showed that ten minutes after the nanosystem was injected into the tail vein, part of it was already distributed in the brain area and maintained for at least 1h.

As these reports have shown, targeting the Tf receptor has been demonstrated to be a good strategy to pass TMZ or other treatments through endothelial BBB cells and improve the accumulation in brain tissues 136, healthy animals' brains 76, and in U87 orthotopic model tumors as well as in TMZ-resistant mouse tumors 135.

Apart from Tf receptors, ***integrin*** receptors are also overexpressed in endothelial cells. Song et al. modified the surface of an NSC with an arginine-glycine-aspartic acid peptide (RGD): a penetrating peptide that facilitates intracellular delivery through integrin receptors 141. They showed that the IC_50_ of cells treated with RGD-functionalized TMZ-NSCs was 10 times lower than cells treated with free TMZ. In addition, the *in vivo* antitumor therapeutic effect was also higher with the nanosystem: mice treated with RGD-TMZ-NSCs had an inhibition rate of 83% compared to control animals, while the rate in animals with nonfunctionalized NSCs was 66% and the one with free TMZ was 21%.

Additionally, a dual-targeting strategy has been reported as a further improvement to TMZ accumulation. A recent study used RGD peptide and lactoferrin to target both integrin and transferrin receptors. This NSC was used to co-deliver TMZ and vincristine 138. They demonstrated better targeting efficacy in the dual-ligand nanosystem than in NSCs targeted with only one ligand. Moreover, tumor inhibition was higher in dual-targeting and both chemotherapies (~ 80%) than in animals treated with only one chemotherapy (around 40%) or one ligand (around 60%) 138.

Targeting Low density ***lipoprotein receptor*** (LDL) has also been demonstrated to be a good strategy for delivering TMZ through BBB 78,142. Seok et al. used liposomes functionalized with the angiopep-2 peptide, an LDL receptor-targeting ligand 142. They showed that mice bearing U87-stem cell tumors had a decreased tumor size; their life span was increased (from 0 in untreated animals up to 211.2%), as was the median survival time (from 23.3 days up to 49.2 days). Recently, Ismail et al. used liposomes functionalized with another LDL receptor-targeting ligand named Apolipoprotein E 143. In this study, they tested liposomes loaded with TMZ and artemisinin, a drug that induces dysregulation of the Wnt/β-catenin pathway, interfering with MGMT and inducing ROS. Hence, they demonstrated in TMZ-resistant U251-bearing mice that the targeting ligand was effective, increasing drug accumulation in the brain. Moreover, they observed that both treatments were efficient in TMZ-resistant tumors, decreasing the tumor size with no observable aggressive tumor growth as happened in control animals. In addition to liposomes, the surface of polymeric micelles has also been functionalized with angiopep-2, demonstrating a higher accumulation of TMZ in the brain and an improved efficacy of the drug, decreasing the tumor size of mice bearing an orthotopic U87 model and improving their survival 78.

As targeting receptors overexpressed in endothelial cells has been demonstrated to be a good strategy for enhancing TMZ brain accumulation, researchers are studying more alternatives. Zhang et al. used glucose attached to liposomes to deliver TMZ and a pro-apoptotic peptide 144. Studies in a highly aggressive intracranial tumor mouse model showed that the system could easily penetrate the BBB (using the glucose-GLU1 pathway), release the two drugs, exhibit a better antitumor effect, and improve the total survival of the animals. Other BBB endothelial-specific transporters have been targeted by short peptides 145. Gabay et al. developed liposomes with these moieties loaded with TMZ, curcumin, and doxorubicin. They demonstrated *in vivo* that functionalized liposomes could cross the BBB and penetrate the brain 35% more than nontargeted liposomes. Furthermore, they showed that tumor growth was delayed and mice survival was increased in animals treated with targeted liposomes plus the three drugs, compared to animals treated with nontargeted liposomes or free drugs 145.

To further know which ligand could be used, Arcella et al. incubated TMZ-loaded liposomes in human plasma and administrated them to human umbilical vein endothelial cells. They studied which proteins attached to liposomes were more concentrated in those cells, observing that liposomes with a higher concentration of molecules, such as apolipoproteins, vitronectin, or vitamin K-dependent proteins, on their surface were accumulating better. Indeed, when the liposomes were added to U87 cell cultures, they showed an enhanced TMZ anti-tumoral effect 149. This preliminary *in vitro* study may serve as the basis for the development of new therapeutic targets.

#### Targeting glioma cells

Tumors are composed of several cell populations, including malignant cancer cells, supportive cells, tumor-infiltrating cells, and cancer stem cells. As mentioned in the previous section, the functionalization of nanosystem surfaces with ligands that target overexpressed receptors of BBB endothelial cells enhances the accumulation of TMZ in the brain tissue. Hence, nanosystem surfaces can be modified to improve TMZ targeting to other types of cells, such as malignant cancer cells or glioma stem cells.

Studies have demonstrated that the active targeting of cancer cells by nanosystem surface modification enhances the efficacy of TMZ. It has been described that there are some receptors overexpressed in cancer cells, including ***transferrin***, ***integrin***, ***folate***, ***EGFR***, ***CD13***, ***monocarboxylate transporter-1*** and ***biotin*** (**Figure 5**).

The expression of the transferrin receptor is increased not only in endothelial cells but also in proliferating cells and cells that have undergone malignant transformation 150,151. Indeed, researchers have modified some nanosystems to target Tf receptors. *In vitro* studies carried out by Ramalho et al. showed that polymeric nanoparticles functionalized with an antibody against the transferrin receptor enhanced TMZ accumulation and efficacy in U251 and U87 cell lines 32. Patil et al. also used anti-transferrin receptor antibodies to target TMZ in cell lines, demonstrating that encapsulated TMZ had a higher efficacy in cell lines resistant to TMZ 139,140. Moreover, Kim et al. designed a liposome functionalized with an antibody anti-Tf receptor and demonstrated a higher uptake of the nanosystem not only *in vitro* in U87, U251, and two TMZ-resistant cell lines, but also confirmed a better TMZ efficacy in mice bearing orthotopic U87 tumors and TMZ-resistant tumors 135.

Another receptor overexpressed not only in endothelial cells but also in glioma cells is the integrin receptor. Studies have demonstrated that mice bearing U87 subcutaneous tumors and treated with NSCs functionalized with RGD peptides efficiently delivered TMZ not only through the BBB but also into glioma cells 138,141.

Folic acid, a molecule responsible for the initiation and propagation of cancer due to its role in cell division and proliferation, has also been attached to nanosystems to deliver TMZ to cancer cells 152. An *in vitro* study showed that polymeric nanoparticles functionalized with folic acid enabled the internalization of TMZ in glioma cells compared to cells that did not have folate receptors overexpressed 146. Moreover, TMZ efficacy was enhanced showing a higher cytotoxic effect in cells overexpressing the receptor 146. Peng et al. functionalized polymeric micelles with folic acid to co-deliver TMZ and an anti-Bcl-2 siRNA 79. They first demonstrated in C6 cells that the functionalization with folic acid improved the delivery of TMZ and the siRNA. Moreover, *in vivo* experiments with rats bearing intracranial C6 cells showed that functionalized micelles decreased by 1.6 times the tumor volume compared to nonfunctionalized micelles 79.

It has also been described that the EGF receptor is overexpressed in glioma cells and approximately 50% of GBM patients present EGFR amplification. Banstola et al. used an anti-EGFR antibody to target polymeric nanoparticles containing TMZ 33. They showed an increase in the cellular uptake of functionalized nanoparticles, together with a higher TMZ efficacy 33. Moreover, Schmitt et al. functionalized chitosan-PLGA polymeric nanoparticles with a small molecule that binds to EGFR, demonstrating that functionalization enhances the TMZ effect in U87 cells 34.

Other less explored targets that have been used to deliver TMZ to GBM cells are the ***CD13*** 60,153, the ***monocarboxylate transporter-1*** 154 (a transporter that regulates the activity of signaling pathways and controls the exchange of monocarboxylate in aerobic glycolysis 155), and ***biotin*** 81 (a molecule whose levels and receptors are highly expressed in tumors 156).

#### Targeting glioma stem cells

Lapidot et al. discovered the first experimental evidence that proved the activity of cancer stem cells in hematological malignancies. They showed that the CD34^+^/CD38^-^ cell subpopulation from acute myeloid leukemia could form leukemia after transplantation into NOD.SCID mice 157. After this study, different researchers have demonstrated the presence of cancer stem cells in other types of cancers, such as breast 158, prostate 159, colorectal 160,161, pancreatic 162 and brain cancers 163-166.

Glioma stem cells, also known as glioma initiating cells, are a small subpopulation of slow-growing cells that exhibit stem cell properties. They have self-renewal and differentiation abilities, which leads to tumor formation and progression 167-169. They present neural stem cell molecular signatures such as Wnt/β-catenin, bone morphogenetic protein, and Sonic Hedgehog, Notch, STAT3, or EGFR pathways 170. Moreover, GSCs are quiescent and have infiltrative capacity, can modulate the microenvironment for their maintenance 171, and are responsible for therapy resistance 172,173. Therefore, the crucial role of GSCs in GBM and their characteristics make them a promising therapeutic target.

It is already known that CD133 is an important cell surface marker for cancer stem cells and also for GSCs 164. Moreover, Singh et al. demonstrated that only a small number of CD133^+^ patient-derived cells were able to initiate tumors in NOD-SCID mouse brains. Indeed, they showed that this CD133^+^ xenograft resembled the original patient tumor. They further observed that these CD133^+^ cells had the capability of self-renewal by isolating those cells from primary tumors and reinjecting them into a secondary mouse. After five weeks they observed that these tumors obtained the phenotype of both the original patient tumor and the primary mouse tumor 165.

Taking this information into account, researchers have recently targeted TMZ specifically to the GSC subpopulation using CD133 markers to improve drug outcomes and drug resistance (**Table 3**). Studies carried out by Smiley 75,147 encapsulated TMZ and idasanutlin in polymeric nanoparticles and conjugated them with an aptamer against CD133. Although they did not observe statistical differences, they noticed a trend of higher killing capacity of CD133-labeled nanoparticles compared to that of nonlabeled nanoparticles. Moreover, the co-delivery of both TMZ and idasanutlin effectively increased the cytotoxicity percentage from ~ 10% with TMZ-loaded nanoparticles up to 80% with TMZ and idasanutlin targeted nanoparticles. A more recent study carried out by Behrooz et al. 74 demonstrated a higher killing capacity of CD133 targeted polymeric nanoparticles together with higher nanoparticle accumulation in U87 stem cells. In this case, they also conjugated an aptamer against CD133 to polymeric nanoparticles, and they co-loaded TMZ with paclitaxel 74. Seok et al. went one step further and demonstrated *in vivo* using U87 GSC-bearing mice that their nanoformulation, composed of liposomes functionalized with an anti-CD133 monoclonal antibody and the angiopep-2 peptide, was better accumulated in the brain, demonstrating an optimal targeting ability and an improved efficacy of TMZ 142. Indeed, the median survival rate of mice was doubled in animals treated with dual-targeted liposomes compared to animals treated with free TMZ. Besides, the anti-tumor efficacy was studied visually by IVIS and the bioluminescence intensity (photon flux) of tumors treated with the nanosystem was 1.000-10.000 times lower than that of free TMZ or nontargeted liposomes 142.

### Local delivery

**Figure 6:**
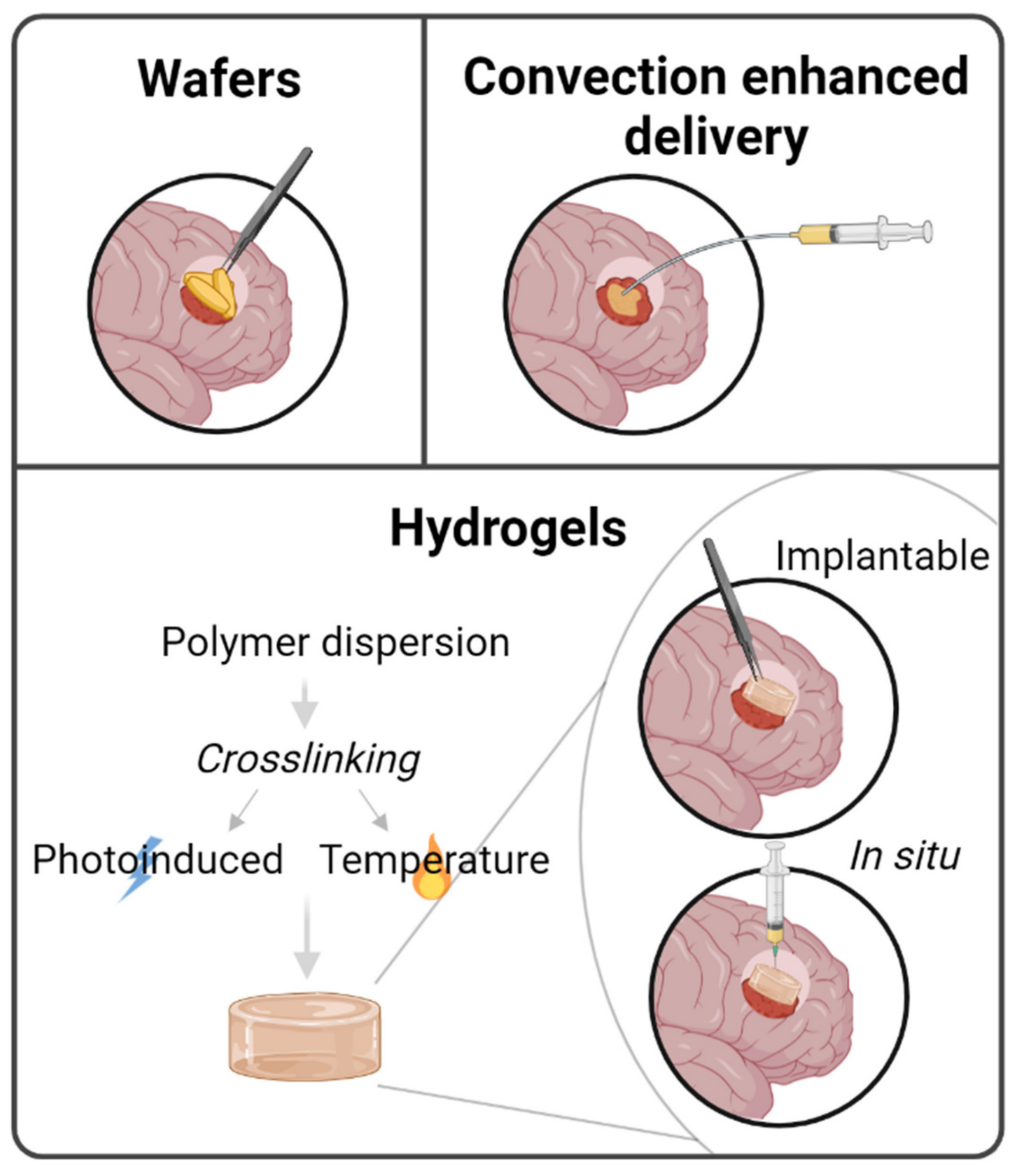
Illustration of local delivery for GBM including implantable wafers, convection-enhanced delivery and hydrogels, where implantable or *in situ*-formed hydrogels can be distinguished.

The use of implants in the case of GBM is minimally invasive because surgery is part of the patients' treatment (65-75% of patients undergo surgical resection 174). Gliadel wafers (loading carmustine) already approved by the FDA, are placed in resection cavities after surgery for 2-3 weeks to prevent recurrence 175. However, its use has been restricted due to its side effects and low drug diffusion. In fact, the stiffness of implants is a key feature: materials stiffer than the brain tissue can enhance gliosis and inflammation, but can also modulate GBM proliferation and invasion. On the contrary, materials softer than the brain tissue have worse stability and fixation at the implant site and are less effective 176. Thus, strategies are being developed for the local delivery of TMZ including hydrogels or convection-enhanced delivery (**Figure 6**). Hydrogels are composed of a three-dimensional hydrophilic polymeric network formed by the crosslinking of polymers in an aqueous medium. Due to their high-water content they are soft and flexible and, hence, have recently attracted much attention. Crosslinking of polymer chains can be achieved by chemical or physical reactions, generating a chemical or physical gel. Hydrogel formation can occur before the local injection (implantable hydrogels) or *in situ* 177, offering the possibility of directly injecting it into either the surgical cavity or the tumor 174. The most studied hydrogels for TMZ encapsulation are those formed *in situ*. For TMZ, the *in situ* crosslinking that has been studied is the photoinduced polymerization, forming irreversible covalent bonds, or via self-assembly in response to temperature 178. Several studies have demonstrated the tolerability and efficacy of ***photoinduced hydrogels*** containing TMZ 179,180. Fourniols et al. 179 designed a hydrogel composed of PEG dimethacrylate (PEG-DMA) that photopolymerized under 400 nm after its injection in the brain cavity. They analyzed the tolerability of the hydrogel by creating a cavity in the cortex of healthy NMRI mice. They evaluated microglial activation and apoptosis in the surrounding brain tissue once the hydrogel was implanted and observed that this activation was induced by brain tissue resection itself, not due to hydrogel implantation. Moreover, microglial activation was not detected inside the hydrogel. Another study carried out by Zhao et al. first encapsulated paclitaxel in PGLA nanoparticles and co-loaded them with TMZ in a PEG-DMA photoinduced hydrogel 180. The injectable pre-hydrogel mixture was photopolymerized under 400 nm light into the resection cavity before closing the cranial window, and they confirmed that U87-bearing mice showed i) no infiltrative cells inside the co-loaded hydrogel and ii) no tumor recurrence after hydrogel implantation (before 110 days post-tumor inoculation). This study also demonstrated that co-delivery of TMZ with another drug enhances the synergic effect of both drugs. Indeed, although the overall survival of mice with just one drug increased 2 and 2.5 times with TMZ and PTX, respectively, compared to untreated mice, animals treated with both drugs were still alive at the end of the experiment (110 days after tumor implantation).

In addition to photoinduced hydrogels, TMZ has been encapsulated in hydrogels that crosslink in response to ***temperature*** changes 181-183. Adhikari et al. 181 used an amphiphilic diblock copolypeptide hydrogel of 180-poly-lysine and 20-poly-leucine that solidifies at room temperature. They tested its efficacy in an orthotopic glioma xenograft mouse model and demonstrated that the hydrogel drastically reduced the tumor size compared to tumors treated with systemically administrated TMZ. In addition, the overall survival rate was increased almost twofold in animals treated with hydrogels compared to those treated with TMZ alone 181.

In addition to *in situ-*formed hydrogels, injectable hydrogels containing TMZ are also being studied. For instance, Zhao et al. 182 have used an injectable hydrogel loaded with TMZ in combination with an MGMT inhibitor that was formed after the mixture reached room temperature. In their study, they generated an orthotopic TMZ-resistant glioma model and showed that the hydrogel i) inhibited MGMT expression and sensitized TMZ-resistant cells to the drug, and ii) reduced tumor growth *in vivo* after surgical resection, proving the efficacy of the hydrogel after partial removal of the tumor. Thermoresponsive hydrogels can also be loaded with nano/microstructures. In fact, Ding et al. 183 encapsulated TMZ in PEG-dipalmitoylphosphatidylethanoiamine calcium phosphate nanoparticles and placed them together with paclitaxel-loaded nanoparticles in a thermoresponsive hydrogel. This system promoted a synergic inhibition effect of the TMZ and PXT nanoparticles-hydrogel increasing twofold the overall survival rate of U87-bearing rats compared to control rats treated with surgery alone. Moreover, they showed that this overall survival increase was accompanied by a higher number of apoptotic cells 183. Furthermore, Akbar et al. 184 designed a hydrogel composed of TMZ, PLGA, and plasticizers that make the material softer. In this case, the hydrogel was implanted 15 days after the tumor removal. Notably, the hydrogel reduced the tumor weight by 98% and 94% in mice and rats after 35 days, respectively. Puente et al. developed an injectable chitosan hydrogel crosslinked with glutaraldehyde 185. Apart from TMZ, this hydrogel contained a radioactive isotope agent (iodine) encapsulated in alginate microparticles for local chemo-radiotherapy treatment of GBM 185. This approach demonstrated for the first time in a GBM subcutaneous mouse model that the tumor size reduction in animals with the hydrogel compared to control animals was due to the localized TMZ and retention of the radioisotope, suggesting that chemo-radio-hydrogel implants might enhance the local control and overall result of GBM. Although in this study the authors did not study the possible effect of the remaining glutaraldehyde, several studies have reported that plain chitosan hydrogels crosslinked with glutaraldehyde and their degradation do not have any toxic effect 186,187, suggesting that the antitumor effect was caused by the cargo.

In addition to the use of hydrogels, convection-enhanced delivery (CED) has also been studied for local delivery of TMZ. The first such study was conducted by Nordling-David et al., who used liposomal TMZ 188. Although in this study there were no statistically significant differences between animals treated with free TMZ and TMZ encapsulated in liposomes, animals treated with the liposomal formulation had a 22% longer survival time. Moreover, they showed a significant decrease in the volume of edemas 188. Also, in a study that used PEGylated liposomes administrated via CED showed that the formulation inhibited tumor growth with no systemic toxicity, suggesting that convection-enhanced delivery could be a good method for the intratumoral administration of TMZ 189.

Although local administration has been less explored in GBM, it offers some advantages, including avoidance of crossing the BBB, access to a drug reservoir near the tumor, improvement in the drug efficiency, and reduction of systemic toxicity.

## Future perspectives

As pointed out in this review, the use of nanosystems for TMZ encapsulation has improved both TMZ limitations and the limitations associated with GBM in *in vivo* models. However, the clinical outcomes of these studies are limited. In this section, we analyze different approaches that can be improved to reduce the time from bench to bedside.

### Improve *in vitro* and *in vivo* models

Most studies presented in this review have been performed *in vitro* in commercial cell lines such as U87, U252, and T98G. These cell lines present enrichment of cancer stem cells when they grow as spheres. However, these classical 2D cellular models are limited due to intrinsic limitations, including inappropriate cell density, gradients of medium components, non-physiological oxygen levels, disruption of the GBM original spatial context, lack of interactions with the extracellular matrix, and lack of other nontumor cells in the GBM microenvironment. Moreover, successive cell passages can select cells with the greatest proliferative potential, decreasing the genetic heterogeneity, and resulting in genetic drift, accumulation of chromosomal aberrations, and phenotypic alterations in cell lines. To improve some of these drawbacks, cell lines derived from surgical samples of GBM patients that preserve GSC features have been developed. Indeed, it is known that GSCs are vital to maintain tumor heterogeneity, as well as tumor initiation, maintenance, and invasion *in vivo* due to their self-renewal and differentiation capacity. Patient-derived GSCs are maintained *in vitro* under floating conditions, as neurospheres, with a serum-free medium supplemented with bFGF and EGF. Nevertheless, neurospheres can be composed of non-homogenous cell populations, the environment can limit stem cell division and induce cell death, and when drugs or treatments are administrated, the distribution can be uneven in the sphere. As GSCs do not need to grow in suspension, GSC laminin-adherent cultures are being used to improve the degree of homogeneity, enhance imaging analysis, and enable clonal propagation. Regardless of the culture conditions (neurosphere or adherent), the GSC model has a major disadvantage: the addition of EGF and bFGF to the culture causes the cells to lose EGFRvIII and EGFR amplification, which is present in approximately 50% of GBM cases. When researchers have tried to grow GSCs without these growth factors, it seems that their survival and maintenance have been affected.

Regarding the *in vivo* models used in the studies mentioned in this work, they are mainly xenograft models in immunodeficient animals with cell lines (U87, U252, and T98G) injected subcutaneously in the flank (heterotopic implantation) or directly in the brain (orthotopic implantation). To improve the outcome of the model and maintain the histopathologic, genomic, and phenotypic characteristic of the primary tumors, patient-derived xenografts might be implemented. Moreover, GSCs could be used to initiate tumors to preserve the tumor heterogeneity better. These models have advantages, including the possibility to test the efficacy of the nanosystems in single patients, the maintenance of the original tumor characteristics, and their genetic stability. However, all these models are done in immunodeficient mice and, thus, they do not have inflammatory responsive cells or an intact immune system. Consequently, the microenvironment and the stroma of the tumor are not the same, limiting the study of the biology and the tumor resistance.

New approaches are being used to study GBM including 3D co-cultures, microfluidics, organoids, and 3D bioprinting 190, which could overcome the disadvantages of above cited models. Hydrogels coated with 3D scaffolds are being used to culture GSCs or pieces of patient-derived cells to create a more realistic tumor environment. These 3D co-cultures that include more than one type of cell, reproduce the cell growth environment of GBM, as well as the soluble signaling and extracellular matrix signaling. Although this model can be useful as a first approach, the substrate stiffness, the selection of the cells across the cell passages, and the constant microenvironment limit this model. Thus, microfluidic devices that can create a changing environment are being developed. However, this technology is still expensive and time-consuming, and although it is an adequate model to study specific targeted drug tests, it takes time to search for appropriate materials to deliver microfluidics depending on the type of study. Although GSCs are the best available *in vitro* model, there are no physiological and reciprocal interactions between GSC and other cells such as nontumor cells and vascular cells. To improve upon these drawbacks, brain organoids are being developed. Brain organoids are 3D tissues generated from pluripotent stem cells, such as induced pluripotent stem cells and embryonic stem cells, or adult-tissue-resident cells, which, in a controlled environment, grow and differentiate slowly. This approach resembles the cell heterogeneity of the tumor microenvironment *in vivo* and it is suitable to study the niche microenvironment and cell invasion. Moreover, cell populations can be genetically modified and, hence, it is possible to study the consequences of genome mutation, evaluate the susceptibility of patients to combinations of driver mutations, and test new therapies and treatments. Although this methodology is limited by the lack of vasculature, immune cells, and BBB functions, it could be very useful to study local delivery systems. In fact, it has been proven in metastatic colorectal cancer that patient-derived tumor organoids predict the response of the biopsied lesion in more than 80% of patients treated with irinotecan-based therapies without misclassifying patients who would have benefited from treatment 191. Thus, brain organoids are a promising new technology for *ex vivo* study of the molecular and cellular mechanisms of the disease, as well as for treatment.

### Synthesis and storage conditions

As shown in section "Improving the solubility and stability of TMZ", the design of nanoformulations is becoming more and more complex, making sample reproducibility and development particularly difficult, which translates into the high cost of nanosystems. To improve reproducibility and decrease the time between preclinical and clinical studies, nanosystems should be designed considering manufacturing constraints in the industry 192. The design of all nanoformulations must ensure their sterility; thus, finding an appropriate sterilization method that does not compromise their physicochemical properties and stability is critical. Another important aspect to consider is the storage condition of the formula. Understanding the effects of storage conditions on the stability and biocompatibility of nanosystems is vital for their clinical application. Storage in water, PBS, or biological fluids may affect the size, surface charge, drug-release profile, and degradation of the nanoformulation. As Abdelwahed proposed, freeze-drying could be evaluated as a universal storage approach 193.

### Study the interaction of the nanosystem with the plasma: Biocorona

Most studies that have designed nanosystems for the encapsulation and delivery of TMZ are administrated systemically. When a nanosystem is administrated through this route, it is covered with plasma proteins and lipids, forming a biocorona. It is already known that the distribution and subsequent pharmacological and toxicological effects depend on this biocorona 194,195. Thus, studying the biocorona *in vitro* and *in vivo* may help predict the biocorona in clinics, thus making it possible to predict the biodistribution and efficacy of the nanosystem in patients. In this sense, Mahmoudi has already determined some strategies that may improve the translation of nanomedicine from preclinical to clinical studies 195. Incubation of a nanosystem in the same protein source (e.g. human plasma) has significant effects on the composition of the biocorona, depending on the age, gender, ethnicity, or health/pathological condition of the donors. Thus, it is important that studies analyzing biocorona consider all this information to predict the behavior of nanosystems in blood circulation.

### Biodegradation of the nanosystems

So as not to limit the use of drug delivery systems, it is important to verify their biodegradation once they have performed their function. In the case of Gliadel wafers, for instance, although they are already approved by the FDA, their use has been restricted due to their side effects. Thus, it is vitally important to verify that the system is biocompatible in the long term and does not generate adverse effects on healthy tissues. Lipid-based nanosystems are probably the most biodegradable and biocompatible nanomaterials because they are usually composed of the same phospholipids as the cell membranes. In the case of polymer-based nanosystems, PLGA nanoparticles might be the most biocompatible. However, as other nanomaterials such as silica nanoparticles or metallic nanoparticles have been less explored, more *in vitro* and *in vivo* studies are needed to analyze their biocompatibility so as to further their move to clinics.

## Conclusions

The incorporation of TMZ into the standard gold treatment of GBM in 2005 was a relevant step forward in extending the overall survival of patients. However, the poor solubility and hydrolyzation of TMZ in physiological media, together with the intrinsic characteristics of GBM have led to an impedance in the effectiveness of GBM therapy.

To overcome these limitations, several nanomaterials to encapsulate TMZ in nanosystems are being postulated, with polymeric and lipid-based nanocarriers being the most advanced ones. It is already demonstrated that TMZ encapsulation increases its solubility, blood circulation, half-life, and biodistribution. Moreover, due to the high surface/volume ratio of nanomaterials, they can be functionalized with ligands to specifically target endothelial cells and enhance BBB crossing and glioma cells. Besides, as it has been described that glioma stem cells could be responsible for the recurrence and resistance of GBM, TMZ has also been specifically targeted at this population of cells using anti-CD133 targeting moieties. Several studies have already demonstrated that this approach is effective for TMZ-resistant tumors *in vivo*.

To reduce the time spent moving from pre-clinics to clinics, some important limitations need to be overcome. It is crucial to improve *in vitro* and *in vivo* models, paying special attention to GSCs, brain organoids, and patient-derived xenografts. Moreover, a lack of information regarding the physico-chemical characterization of the designed nanosystems is still a factor. This causes problems particularly in sample reproducibility and the development of a robust, scalable, and affordable preparation process; thus, it is crucial to pay special attention to sterility and the storage conditions of the nanoformulations. Another important aspect to consider is the interaction of the nanocarrier with the biological context: first with the blood circulation if the chosen route is the systemic one, and then with the interaction of the nanosystem with the specific cell. Thus, more studies are needed to understand the specific nanosystem/cell interactions, paying special attention to the nanomaterial used (size, surface charge, and shape) and the *in vitro* or *in vivo* models.

In summary, some unknowns still remain to be overcome. However, nanosystems can improve the delivery and efficacy of TMZ, suggesting that this strategy is promising in the field of nanomedicine and oncology, particularly in GBM.

## Figures and Tables

**Table 1:** Advantages and disadvantages of the different nanosystems that have been used to improve TMZ limitations based on polymers, lipids, and other nanomaterials.

		Advantages	Disadvantages
**Polymer-based nanosystems**	Polymeric nanoparticles	Scalable production, improved TMZ stability, co-encapsulation	Low encapsulation efficiency
	Dendrimers	Improved TMZ stability, controlled release, multiple encapsulation, co-encapsulation	Reduced number of studies
**Lipid-based nanosystems**	Liposomes	More studied nanosystem, excellent biocompatible, sustained release, multiple encapsulation, co-encapsulation	Rapid clearance, low stability, immune response, not studied with other drugs
	Solid lipid nanoparticles	Sustained release, stability, scalable industrial production	Reduced number of studies, not studied with other drugs
	Nanostructured carriers	Biocompatible, encapsulation efficiency, controlled release, co-encapsulation	Reduced number of studies
**Other nanomaterials**	Mesoporous silica nanoparticles	Biocompatible controlled release, co-encapsulation, combination with other nanomaterials	Reduced number of just *in vitro* studies
	Graphene oxide nanoparticles	Improved TMZ efficacy	There is only one *in vitro* study
	Magnetic nanoparticles	Improved TMZ stability and cell uptake, improved TMZ efficacy, co-encapsulation	There is only one *in vitro* study

**Table 2:** Nanosystems that have been studied for TMZ co-delivery with other molecules.

Nanosystem		Co-delivery	Therapeutic strategies	Studies	Ref
Polymer-based nanosystems	Polymeric nanoparticles	Doxorubicin	Chemotherapy	Characterization studies	37
		5-Fluoracil	Chemotherapy	Characterization studies	39
		Idasanutlin	MDM2 inhibitor	*In vitro* (GSCs)	75
		si-EGFR	Nucleic acids	*In vivo*	77
		Super-paramagnetic nanoparticles	Magnetic hyperthermia	*In vitro*	85
		Cy5-dye	Imaging	*In vivo*	34
	Dendrimers	Paclitaxel	Chemotherapy	*In vitro*	74
	Polymeric micelles	si-Polo like kinase 1	Nucleic acids	*In vivo*	78
		Si-Bcl2	Nucleic acids	*In vivo*	79
		Verteporfin	Photodynamic therapy	*In vitro*	81
Lipid-based nanosystems	SLNPs	Vincristine	Chemotherapy	*In vitro*	55
		Magnetic nanoparticles	Magnetic hyperthermia	*In vitro* (spheroids)	86
	Liposomes	Bromodomain inhibitor	Inhibitors	*In vivo*	76
		Super-paramagnetic nanoparticles	Magnetic hyperthermia	*In vitro*	87
	NSCs	Vincristine	Chemotherapy	*In vitro*	55
		GFP gene	Nucleic acids	*In vivo*	80
		Curcumin	Other molecules	*In vivo*	88
Other nanomaterials	Silica-based nanoparticles	anti-miR221	Nucleic acids	*In vitro*	58
		Porous silicon NPs	Photothermal therapy	*In vitro*	83
		DNAzyme	Other molecules	*In vitro*	59
	Magnetic nanoparticles	Indocyanine green	Photothermal and photodynamic therapy	*In vitro*	82
		Curcumin	Other molecules	*In vitro*	62
	Copper-based nanoparticles		Photothermal and photodynamic therapy	*In vitro*	84

**Table 3:** Most studied targeting strategies to improve TMZ delivery to the cells of interest.

Ligand	Target	Nanosystem	Moiety	Advantages	Ref
**Tf**	BBB Glioma cells	Liposome PEGylated liposomes SLNPs Polymeric NPs	Antibody Aptamers Peptides	*In vitro, in vivo* studies Dual targeting Co-delivery Improve TMZ resistance	32,76,135-140
**Integrin**	BBB Glioma cells	NSC	Penetrating peptide	*In vitro*,* in vivo* studies Dual targeting	141 138
**LDL**	BBB	Liposomes Polymeric micelles	Peptides Protein	*In vitro, in vivo* studies Co-delivery	78,142,143
**Transporters**	BBB	Liposomes	Glucose Peptides	*In vitro, in vivo* studies Co-delivery	144,145
**Folate**	Glioma cells	Polymeric NPs Polymeric micelles	Folic acid	*In vitro, in vivo* studies	79,146
**EGFR**	Glioma cells	Polymeric NP	Antibody Small molecule	*In vitro* studies	33
**CD133**	GSCs	Polymeric NPs Liposomes	Aptamer Antibody	*In vitro, in vivo* studies Dual targeting Co-delivery	74,75,142,147
